# Need to Belong and Social Ties in Late Life

**DOI:** 10.1093/geroni/igab046.3323

**Published:** 2021-12-17

**Authors:** Zexi Zhou, Yijung Kim, Shiyang Zhang, Karen Fingerman

**Affiliations:** The University of Texas at Austin, Austin, Texas, United States

## Abstract

According to socioemotional selectivity theory, older adults are more selective and tend to shrink their social network to their closest ties. However, a heightened need to belong, which is characterized by a stronger desire for acceptance and motivation to affiliation, may alter this common pattern. We know little about how the need to belong shapes social network structure, especially in late life. This study investigated the associations between the need to belong, size of social network, and engagement with social ties among older adults. Participants (N = 314) aged over 65 years from the Daily Experiences and Well-being Study completed a baseline interview regarding their close ties (i.e., social convoy members), and weak ties, as well as a self-report measure of need to belong. They completed ecological momentary assessment (EMA) surveys reporting their social encounters every 3 hours over 5 to 6 days. Need to belong was unrelated to the number of close ties. In contrast, participants with a higher need to belong reported more less close (but still important) ties and weak ties than those with a lower need to belong, but spent a similar amount of time (i.e., proportion of EMA involving social encounters) with either their close ties or weak ties. These results suggest that the need to belong may motivate older adults to go beyond their closest ties to weaker ties, and highlight the discrepancies between the sense of being connected to social partners and the actual engagement with them in this process.

